# Gut Dysbiosis Has the Potential to Reduce the Sexual Attractiveness of Mouse Female

**DOI:** 10.3389/fmicb.2022.916766

**Published:** 2022-05-23

**Authors:** Xianfeng Yi, Muha Cha

**Affiliations:** ^1^College of Life Sciences, Qufu Normal University, Qufu, China; ^2^Academy of Agricultural Sciences, Chifeng University, Chifeng, China

**Keywords:** antibiotic, gut dysbiosis, gut microbiota, sexual attractiveness, mate preference

## Abstract

Increasing evidence has shown that the gut microbiome has significant effects on mate preferences of insects; however, whether gut microbiota composition affects sexual attractiveness and mate preference in mammals remains largely unknown. Here, we showed that antibiotic treatment significantly restructured the gut microbiota composition of both mouse males and females. Males, regardless of antibiotic treatment, exhibited a higher propensity to interact with the control females than the antibiotic-treated females. The data clearly showed that gut microbiota dysbiosis reduced the sexual attractiveness of females to males, implying that commensal gut microbiota influences female attractiveness to males. The reduced sexual attractiveness of the antibiotic-treated females may be beneficial to discriminating males by avoiding disorders of immunity and sociability in offspring that acquire maternal gut microbiota *via* vertical transmission. We suggest further work should be oriented to increase our understanding of the interactions between gut microbiota dysbiosis, sexual selection, and mate choice of wild animals at the population level.

## Introduction

It is well accepted that all multicellular organisms, including animals and plants living in a world dominated by microbes, harbor a diversity of microbial communities in and on their bodies (Ley et al., 2008; Fierer and Lennon, 2011; Li et al., 2019; Wan et al., 2021). It is suggested that microbiomes can act as an integral part of the host phenotype, or even potentially the genome of hosts (Degnan, 2014; Kolodny and Schulenburg, 2020; Ma et al., 2021). Gut microbes are believed to affect a wide spectrum of host immune and neurological systems and thus play a critical role in most animal life (Arentsen et al., 2015; Gao et al., 2018; Nyangahu et al., 2018; Blacher et al., 2019; Li et al., 2021). Mounting evidence has shown that these microbiomes contained in the gastrointestinal tract can have either detrimental or beneficial impacts on many aspects of physiology, such as the immune and endocrine systems, spanning a continuum influence on host biology (Geva-Zatorsky et al., 2017; Chu et al., 2019). In recent years, the gut microbiota and its interactions with host physiology and immune function have been identified as having a crucial role in the normal development of behaviors (Ezenwa et al., 2012; Leftwich et al., 2017; Parfrey et al., 2018; Bai et al., 2021).

Over the past several decades, rapid advances in molecular methods have greatly improved our understanding of the importance of gut microbiota (Bäckhed et al., 2015; Wada-Katsumata et al., 2015; Tamburini et al., 2016). Gut microbiota, through interacting with the host, can produce intermediate or end products of microbial metabolism, for example, secondary bile acid and short-chain fatty acids (SCFAs; Gao et al., 2018; Zhuang et al., 2019; Zhang et al., 2020). A growing body of evidence has shown that signals from these small molecules derived from bacterial metabolism have prominent structural and functional effects on the development and function of the immune, metabolic, endocrine, nervous, and fitness-related behaviors, such as mating and social interactions (Cryan and Dinan, 2012; Foster and Neufeld, 2013; Bäckhed et al., 2015; Palmer et al., 2017).

Commensal gut bacteria in adult animals not only protect the host from infection and inflammation of the intestines and periphery but also modulate normal behavioral responses. Previous studies of insects have provided some clear evidence of the potentially profound effect of the gut microbiota on behaviors (Dillon et al., 2000; Wada-Katsumata et al., 2015). Increasing evidence indicates that the gut microbiome of *Drosophila* can have significant effects on mate preferences as well as the mating performance of males and females (Lizé et al., 2014; Najarro et al., 2015; Walsh et al., 2017; Heys et al., 2020). Leftwich et al. (2017) showed that gut microbiomes also have a strong potential to influence reproductive barriers between *Drosophila* populations. Therefore, it can be expected that gut microbiomes will influence the intensity of sexual selection given that gut microbiome increases or decreases mating activity. Despite the importance of gut microbiota in host biology, relatively little is known about the microbial communities in mate choice of males and females. Although the gut microbiome can have major influences on the host mating behavior of insects, there are limited empirical research on the influence of gut microbiota dysbiosis on sexual attractiveness and mate choice of mammals. Therefore, understanding the mechanisms underlying gut microbiome and mating behavior interaction will provide new insight into the symbiotic relationship between gut microbiota and their mammalian hosts. However, sexual dimorphism in response to broad-spectrum antibiotics has been observed in laboratory mice (Fujisaka et al., 2016). It was shown that antibiotics can change the body mass of mice due to changes in gut microbiota (Miao et al., 2019). To avoid possible confounding effects of body mass of males on the mating preference of females, we tested the sexual attractiveness of female mice to males using low-dose oral administration of a combination of broad-spectrum antibiotics vancomycin and neomycin sulfate and showed that gut dysbiosis potentially reduced sexual attractiveness of mouse female.

## Materials and Methods

### Mice and Antibiotic Administration

The adult Kunming mice (KM, 8 weeks of age) were purchased from Jinan Pengyue Experimental Animal Breeding Co. Ltd. (Shandong, China), where the specific pathogen-free animals were housed by litter and allowed access to autoclaved mouse chow water. After purchase, mice were caged individually in a specific pathogen-free facility and reared in a 25°C room on a 12-h light/dark cycle. Mice received standard rat chow (4% fat, 20% protein, 70% carbohydrate, manufactured by Shenyang Maohua Biotechnology Co. Ltd., Liaoning, China) and regular drinking water was provided *ad libitum*. One week after acclimation, 10 males and 10 females were randomly selected and orally administrated with vancomycin (1 mg/mL) and neomycin sulfate (5 mg/mL) as broad-spectrum antibiotics in regular drinking water for 10 days (treatment group), a duration threshold suggested by previous studies (Hill et al., 2010; Swann et al., 2011). Nothing else was added to the regular drinking water except for vancomycin and neomycin sulfate. The remaining 10 males and 10 females were assigned to the control group and continually received regular drinking water without vancomycin and neomycin sulfate. The control individuals were genetically comparable to the individuals in the treatment group because all mice were at the same age before purchase. At the end of the antibiotic administration, the treated and control mice (10-week-old) were subjected to sexual attractiveness tests in the three-chamber test apparatus in the same way. After the behavioral test, all mice were individually weighed and then sacrificed by a neck bite to collect cecal samples approved by the Animal Experimentation Ethics Committee of Qufu Normal University (2022028). Cecal samples were snap-frozen in liquid nitrogen and then immediately transferred to a −80°C refrigerator for subsequent gut microbiota analysis.

### DNA Extraction

A DNeasy PowerSoil Kit (Qiagen, Hilden, Germany) was used to extract the total genomic DNA of fecal samples in OE Biotech Co., Ltd. (Shanghai, China) following the manufacturer’s instructions with a blank extraction control being included to check for any microbial contamination. Cecal samples were randomized across DNA extraction batches to avoid confounding biological and technical effects concentration and purity of DNA were verified with NanoDrop 2000 spectrophotometer (Thermo Fisher Scientific, Waltham, MA, United States) and agarose gel electrophoresis, respectively. The genome DNA was used as a template for PCR amplification with the barcoded primers and Tks Gflex DNA Polymerase (Takara). Moreover, samples were also randomized across PCR plates and sequencing lanes. V3-V4 variable regions of 16S rRNA genes were amplified with adaptors-linked universal primers 343 F (5′-TACGGRAGGCAGCAG-3′) and 798 R (5′-AGGGTATCTAATCCT-3′) (Zhou et al., 2021).

### Bioinformatic Analysis

In our study, sequencing was performed on an Illumina Miseq with two paired-end read cycles of 300 bases each (Illumina Inc., San Diego, CA, United States; OE Biotech Co., Ltd., Shanghai, China). Raw sequencing data were in FASTQ format. Paired-end reads were then preprocessed using Trimmomatic software (Bolger et al., 2014) to detect and cut off ambiguous bases (N). Low-quality sequences with an average quality score below 20 were cut off using the sliding window trimming approach. After trimming, paired-end reads were assembled using FLASH software (Reyon et al., 2012). Parameters of assembly were 10 bp of minimal overlapping, 200 bp of maximum overlapping, and 20% of maximum mismatch rate. Further quality control included removing reads that were ambiguous, identical, or below 200 bp in length. Reads with 75% of bases above Q20 were retained. Then, reads with chimera were detected and removed. These two steps were achieved using QIIME (Quantitative Insights into Microbial Ecology) software (version 1.8.0) (Caporaso et al., 2010).

Clean reads were subjected to primer sequences removal and clustered to generate operational taxonomic units (OTUs) using Vsearch software with a 97% similarity cutoff (Rognes et al., 2016) to generate an OTU table with the taxonomy and number of sequences per OTU in each sample. The representative read of each OTU was selected using the QIIME package. All representative reads were annotated and blasted against Silva database Version 123 (16s rDNA) using the RDP classifier (confidence threshold was 70%) (Wong et al., 2017).

We summarized the rarified OTU table in QIIME (Segata et al., 2011) to see the effects of antibiotic treatment on the community richness and community diversity of cecal samples. The Wilcoxon rank-sum test was used to identify differences in the alpha-diversity of the gut microbiota using community richness (e.g., Shannon index, Simpson index, observed-species, and Chao 1 index) between the antibiotic-treated animals and controls (Chao and Bunge, 2002). The OTU richness was determined by calculating the observed species, Shannon, Simpson, and Chao1 indices based on the total number of species.

We used a variance stabilizing transformation of arcsine (abundance 0.5) to normalize the relative abundances of microbial genera (Ho et al., 2019). Community structure (β-diversity) of the Bray Curtis dissimilarity was generated from QIIME. PCoA and distance matrices were used to analyze the bacterial community data with PRIMER 7 software. Analysis of molecular variance (Adonis) was done on the presence data to test the differences in gut microbiota compositions. The statistical significance was set as P < 0.05. The algorithm of Linear Discriminant Analysis Effect Size (LEfSe) was applied to recognize relative abundant values of OTUs as well as pathways exhibiting significant deviations which would be subjected to a defaulted cutoff in accordance with ranking of the Kruskal–Wallis test (P < 0.05 and the score of absolute log_10_ LDA). GraphPad Prism 9 was used to plot α-diversity results and gut microbiota at phylum and genus levels. An independent samples *t*-test was used to compare the differences in gut microbiota at phylum and genus levels. We used the Phylogenetic Investigation of Communities by Reconstruction of Unobserved States (PICRUSt) v1.0.0 to predict the composition of known gut microbial gene functions based on the KEGG (Kyoto Encyclopedia of Genes and Genomes) and COG (Cluster of Orthologous Groups) database (Douglas et al., 2018).

### Test of Sexual Attractiveness

In this study, we used three-chamber social test apparatus to measure the sexual attractiveness of females to males (Supplementary Figure 1). Sexual attractiveness in our study was defined as the propensity of a male to interact with each of the paired unfamiliar females in the two opposite cages. We chose the male mice as choosers because their body mass was significantly reduced by antibiotics in our study. The apparatus used in this study was made of a polymethyl methacrylate box (length × width × height: 60 cm × 30 cm × 60 cm) with partitions that separate the box into three identical chambers (Supplementary Figure 1). The doors on the partitions, when opened, allowed the test animal to move freely from one chamber to another. At the phase of habituation, the test male mouse was placed in the middle chamber and allowed to move freely in all three chambers for 5 min. After this 5-min habituation phase, the male mouse was confined in the center chamber by closing the doors. Then, an unfamiliar control female was placed inside a small wire cage centered in one of the side chambers, meanwhile, an unfamiliar antibiotic-treated female was placed inside the identical wire cage in the opposite chamber. The doors were then reopened, allowing the test animal to move freely throughout all three chambers of the apparatus for over 5 min. The thin, widely spaced bars of the wire cage allowed us to monitor whether the male mouse initiated social interaction with the two females. To prevent interference between tests, the three chambers and the two-wire cages used for behavioral tests were thoroughly sterilized and cleaned by using 75% alcohol and absorbent cotton. To avoid chamber bias, locations of the two-wire cages were randomly exchanged between left and right chambers on consecutive tests. Measures were taken of entries between chambers, travel distance in each chamber, and time spent sniffing each wire cage containing the unfamiliar female mice on the opposite side of the apparatus using the Any-maze video tracking system from Stoelting Co. (version 6.0, Wood Dale, IL, United States). In our study, 10 antibiotic-treated males and 10 controls were tested individually only once. Here, five paired antibiotic-treated and control females were randomly chosen to interact with the antibiotic-treated males and another five pairs were used to interact with the control males. Therefore, each paired female mice were used repeatedly twice with the discriminating males (Supplementary Table 1). But no female was used repeatedly for the same male. Therefore, a paired *t*-test was used to test if males prefer the control over antibiotic-treated females for sexual attractiveness.

## Results

**FIGURE 1:**
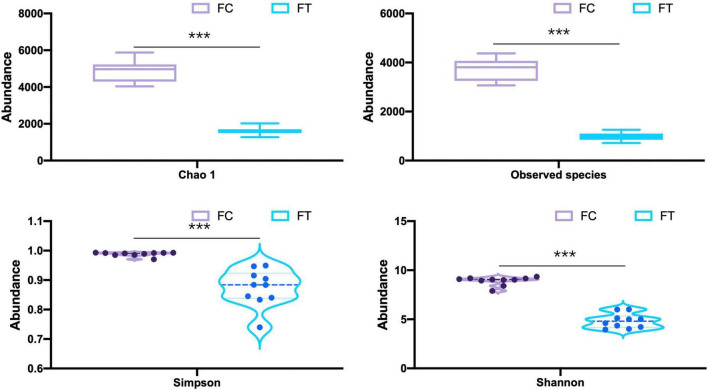
Comparison of the α-diversity indices (Chao 1, the number of species, Shannon, and Simpson) of the control females (FC) and the antibiotic-treated females (FT). Statistical significance: ***, *P* < 0.001.

**FIGURE 2:**
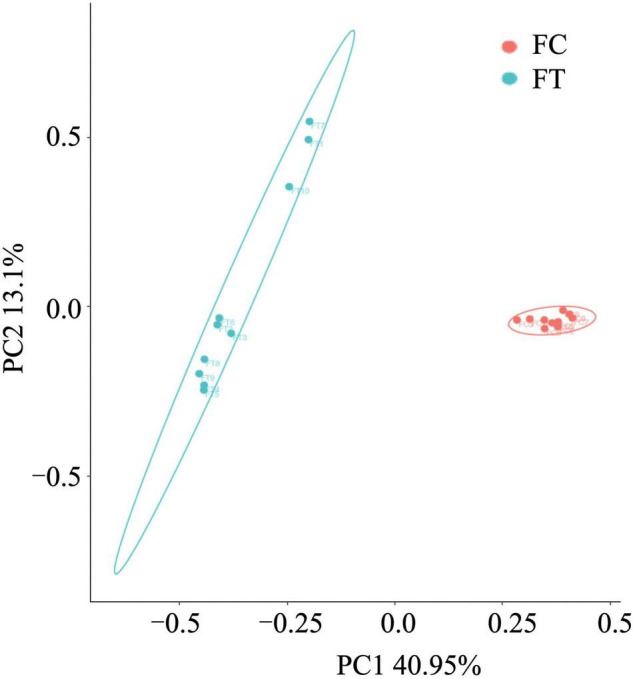
Principal-coordinates analysis (PCoA) based on Bray-Curtis dissimilarities of the gut microbiota in the control females (FC) and the antibiotic-treated females (FT).

We showed that oral administration of antibiotics had profound effects on gut microbiota of both mouse females and males. Gut microbiota α-diversity was decreased in antibiotic-treated females compared to controls, as indicated by Chao 1, the number of species, Shannon, and Simpson (*t* = 16.542, df = 18, P < 0.001; *t* = 18.552, df = 18, P < 0.001; *t* = 15.026, df = 18, P < 0.001; *t* = 5.719, df = 18, P < 0.001; Figure 1). Principal coordinate analysis (PCoA) of β-diversity (by Bray-Curtis dissimilarity) demonstrated that the antibiotic-treated females clustered separately from the control counterparts (Figure 2; Adonis: R^2^ = 0.3973, *P* < 0.001). Antibiotic administration significantly changed α- (Supplementary Figures 2a–d) and β-diversity of gut microbiota of males (Supplementary Figure 2e).

**FIGURE 3:**
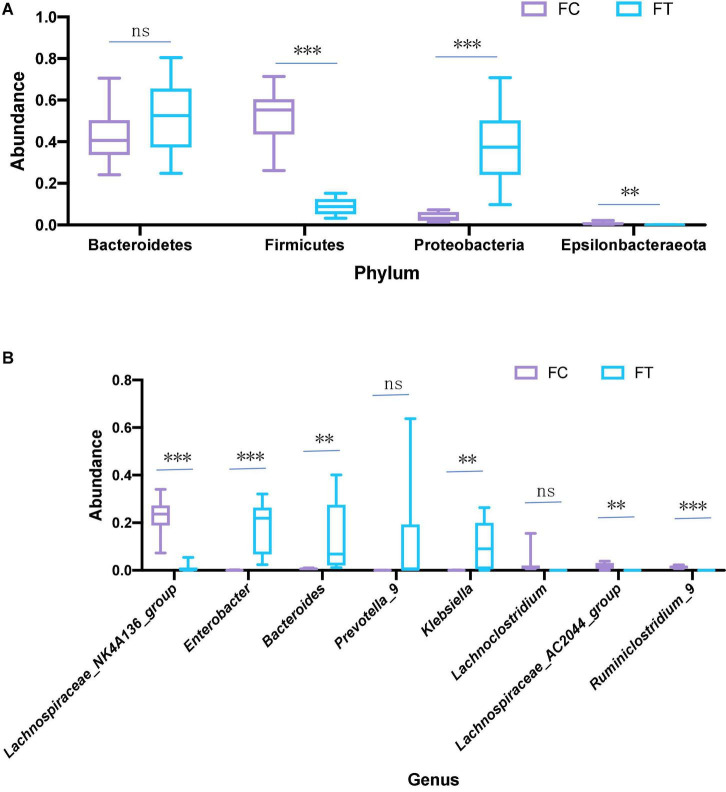
Relative abundance of main phyla **(A)** and genera **(B)** that were significantly different in the gut microbiota in the control females (FC) and the antibiotic-treated females (FT). Statistical significance: ^**^, *P* < 0.01; ^***^, *P* < 0.001; ns, not significant.

**FIGURE 4:**
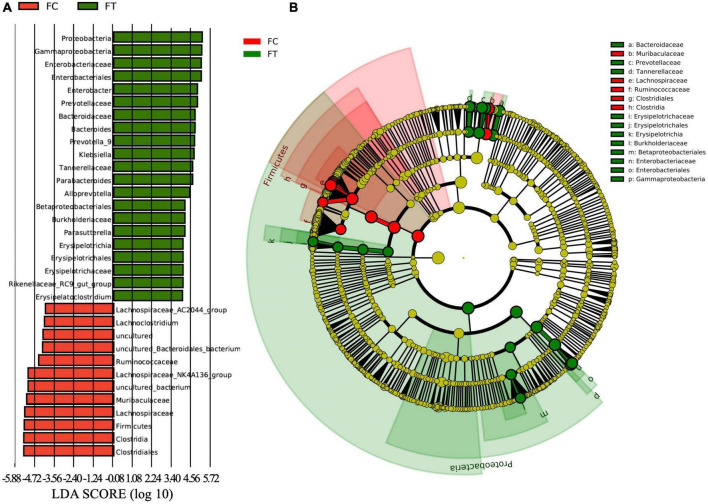
The LDA (linear discriminant analysis) score **(A)** and the taxonomic cladogram **(B)** were obtained from linear discriminant analysis effect size (LEfSe) analysis of the gut microbiota of control females (FC) and the antibiotic-treated females (FT). Bar chart showing the log-transformed LDA scores of bacterial taxa identified by LEfSe analysis (the log-transformed LDA score of 6.0 as the threshold). Cladogram showing the phylogenetic relationships of bacterial taxa revealed by LEfSe. From inside to outside, the circle of radiation represented the classification level from phylum to genus. The red and green nodes in the phylogenetic tree represent gut microbiota that plays an important role in the FC and FT groups, respectively. While yellow nodes represent species showing no significant difference.

At the phylum level, the relative abundance of Firmicutes and Epsilonbacteraeota was significantly decreased in the antibiotic-treated females compared to controls (independent samples *t*-test: *t* = 9.958, *df* = 18, *P* < 0.001; *t* = 3.601, *df* = 18, *P* = 0.002; Figure 3A and Supplementary Figure 3a). The antibiotic-treated females had higher abundance of Proteobacteria than the control group (independent samples *t*-test: *t* = −5.668, *df* = 18, *P* < 0.001). Meanwhile, antibiotic administration showed no significant effect on the relative abundance of Bacteroidetes in females (independent samples *t*-test: *t* = −1.407, *df* = 18, *P* = 0.176; Figure 3A). At the genus level, the antibiotic-treated females had lower relative abundance of *Lachnospiraceae*_NK4A136_group, *Lachnospiraceae*_AC2044_group, and *Ruminiclostridium 9* (independent samples *t*-test: *t* = 8.875, *df* = 18, P < 0.001; *t* = 4.111, *df* = 18, *P* = 0.001; *t* = 8.314, *df* = 18, *P* < 0.001, respectively; Figure 3B), but higher relative abundance of *Enterobacter*, *Bacteroides*, and *Klebsiella* (independent samples *t*-test: *t* = −5.260, *df* = 18, P < 0.001; *t* = −3.063, *df* = 18, *P* = 0.007; *t* = −3.284, *df* = 18, *P* = 0.004, respectively; Figure 3B and Supplementary Figure 3b). These patterns were well reflected in the male mice (Supplementary Figure 4). LEfSe analysis showed that the relative abundance of Lachnospiraceae, Ruminococcaceae, and Clostridiales was significantly increased in the control females. Meanwhile, the relative abundance of Enterobacteriaceae and Gammaproteobacteria was increased in antibiotic-treated females (Figures 4A,B).

**FIGURE 5:**
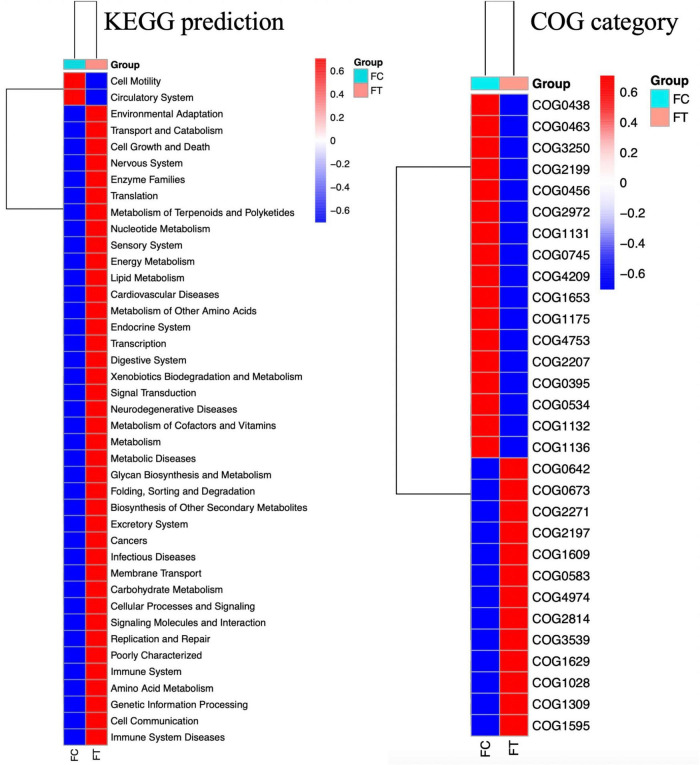
Heat map showing the predicted KEGG and COG functional pathways differing in the gut microbiota of the control females (FC) and the antibiotic-treated females (FT) inferred from 16S rRNA gene sequences using PICRUSt. Red legends indicate the mean of significantly enriched pathways and blue the depleted pathways across all gut samples of mice (*P* < 0.05).

We performed KEGG and COG analyses to further understand the changes in gut microbial function and metabolic activity between antibiotic-induced females and controls. Compared to the control group, infectious diseases (*t*-test: *P* = 0.001), cancers (*t*-test: *P* = 0.001), immune system diseases (*t*-test: *P* = 0.001), cardiovascular diseases (*t*-test: *P* = 0.002), and metabolic diseases (*t*-test: *P* = 0.001) were significantly upregulated in the antibiotic-treated females (Figure 5). However, apparent suppression of circulatory system (*t*-test: *P* = 0.01) was clearly observed in the antibiotic-treated females (Figure 5).

**FIGURE 6:**
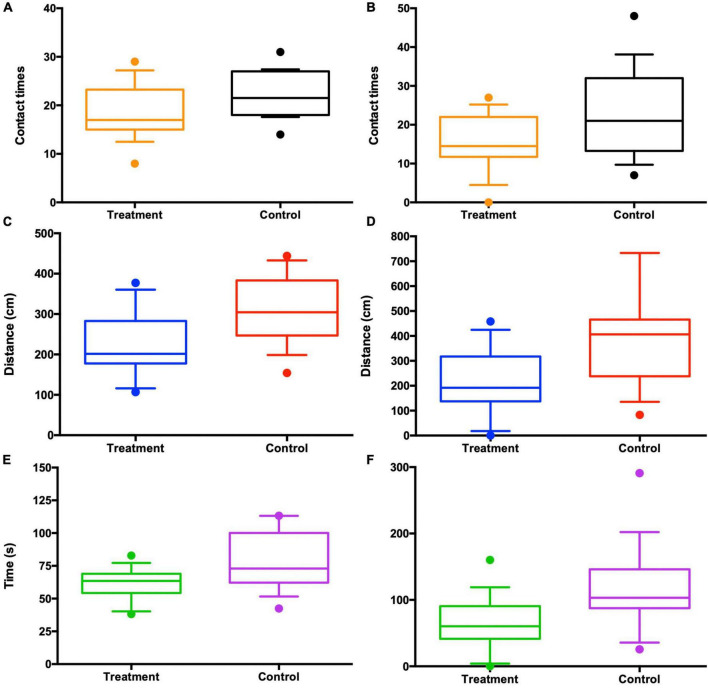
Antibiotic-treated females lost the attractiveness to both control (*n* = 10) and antibiotic-treated males (*n* = 10) in the three-chambered sociability test, as seen from the contact times with the females **(A,B)**, the travel distances in each chamber **(C,D)**, and the time spent in each chamber **(E,F)**. “Control” in the *x*-axis appears to be control females and “Treatment” antibiotic-treated females chosen by males.

In the test sessions for sexual attractiveness, the number of entries of control males into the chambers containing the control females was significantly more than the number of entries into the chambers containing the antibiotic-treated females (paired *t*-test: *P* = 0.048; Figure 6A). Travel distance in the chambers containing the control females was significantly more than the distance in the chambers containing the antibiotic-treated females (paired *t*-test: *P* = 0.003; Figure 6C). In addition, the amount of time spent by control males sniffing the wire cage containing the control females was significantly more than the time spent with the antibiotic-treated females (paired *t*-test: *P* = 0.004; Figure 6E). The antibiotic-treated males performed in the same way as controls, that is, the number of entries into the chambers containing the control females was significantly more than the number of entries into the chambers containing the antibiotic-treated females (paired *t*-test: *P* = 0.019; Figure 6B). Distance traveled in the chambers containing the control females was significantly more than distance traveled in the chambers containing the antibiotic-treated females (paired *t*-test: *P* = 0.021; Figure 6D). Time spent sniffing the wire cage containing the control females was significantly more than the time sniffing the wire cage containing the antibiotic-treated females (paired *t*-test: *P* = 0.013; Figure 6F).

## Discussion

Antibiotics have been well used to alter gut microbiota composition for behavior test purposes (Desbonnet et al., 2015; Ray et al., 2021). Consistent with previous studies (Desbonnet et al., 2014, 2015), our results revealed that antibiotic treatment significantly restructured the gut microbiota composition of both mouse males and females. Overall, we showed that the antibiotic-treated mice had a lower relative abundance of beneficial bacteria phyla, for example, some microbiota belonging to Firmicutes and Epsilonbacteraeota versus controls. While, the relative abundance of Proteobacteria, which is a potential diagnostic criterion for gut microbiota dysbiosis (Nyangahu et al., 2018), was increased in the antibiotic-treated mice compared to controls. Short-term antibiotic treatment of adult mice can lead to immune suppression, while in early life prenatal antibiotic treatment causes contact hypersensitivity or immune development later in life (Strzępa et al., 2017; Gao et al., 2018; Nyangahu et al., 2018). Therefore, the increased abundance of Proteobacteria and possibly the alteration of immune response caused by gut dysbiosis could potentially reduce the sexual attractiveness of antibiotic-treated females (Ueyama et al., 2015).

Host microbiota plays a crucial role in determining sexual attractiveness and mating preference (Lizé et al., 2014; Walsh et al., 2017). However, most studies emphasized the important role of gut microbiota in mate preferences in a number of species of *Drosophila* (Markov et al., 2009; Sharon et al., 2010; Najarro et al., 2015; Heys et al., 2020). Tackling the importance of gut microbiota on social odor and sexual attractiveness of vertebrates is still rare (Theis et al., 2013). After controlling for the potential effect of body mass on mating preference (Independent *t*-test: *t* = −2.398, df = 18, *P* = 0.046; Supplementary Figure 5), sexual attractiveness tests in our study showed that both control males and antibiotic-treated males exhibited higher propensity to interact with control females than antibiotic-treated females, which was reflected by the facts that (1) males preferred to enter into the chambers containing the control females over those containing the antibiotic-treated females; (2) males spent more time sniffing the control females than the antibiotic-treated females; and (3) males traveled more in the chambers containing the control females than containing the antibiotic-treated females (Figure 6). Therefore, the data presented here clearly showed that the antibiotic-treated females exhibited reduced sexual attractiveness to males compared to their control counterparts, suggesting that the influence of the gut microbiota may extend to the modulation of mouse sociality (Desbonnet et al., 2015; Münger et al., 2018; Wu et al., 2021).

Despite gut microbiota dysbiosis, the antibiotic-treated males, like the control males, consistently preferred the control females over the antibiotic-treated females. It has been evidenced that antibiotics can cause body weight loss (Miao et al., 2019); however, we can rule out the potential influence of body weight on the sexual attractiveness of females to males, because antibiotic treatment did not significantly change the body weight of female mice in our study (Independent *t*-test: *t* = 0.357, df = 18, *P* = 0.718; Supplementary Figure 5). These observations suggest that antibiotic-induced gut microbiota dysbiosis strikingly modified gut bacterial composition and then reduced the sexual attractiveness of females to males. We also provided some evidence on the correlation between gut microbiota dysbiosis and sexual attractiveness of females. For example, the relative abundance of Lachnospiraceae, Ruminococcaceae, and Clostridiales, which are generally linked to the production of short-chain fatty acids (Koh et al., 2016; Gao et al., 2018) and play important roles in maintaining the stability of the intestinal environment, was significantly decreased in the antibiotic-treated females that exhibited reduced sexual attractiveness. However, the relative abundance of inflammation-associated microbiota Enterobacteriaceae and Gammaproteobacteria (Artwohl et al., 2000; Garrett et al., 2010; Huffnagle et al., 2017) was decreased in the control females that were more attractive to males. In addition, the relative abundance of propionate-produced Muribaculaceae bacterial family was much higher in the control females, which has been supposed to play a critical metabolic role in increasing the life span (Smith et al., 2019). Thus, it is likely that decrease in the beneficial bacteria but an increase in pathogen-like bacteria may be associated with the disrupted immunity and reduced health level of the antibiotic-treated females (Desbonnet et al., 2014). Therefore, it can be expected that gut microbiota dysbiosis reduces the sexual attractiveness of the antibiotic-treated females possibly by altering the neurological system, immune system, and endocrine system that may have been noticed by males (Strzępa et al., 2017; Wu et al., 2021). This speculation can also be supported by significant suppression of the circulatory system and upregulation of disease-related metabolic pathways in the antibiotic-treated females as predicted by KEGG and COG (Figure 5). Both the control males and the antibiotic-treated counterparts were likely able to detect the differences in health conditions and immune response between the control females and the antibiotic-treated females. Although we are unable to uncover a specific mechanism by which antibiotic treatment abolished mating preference, sex pheromones that determine individual scents are expected to contribute to a decrease in female attractiveness to males influenced by the gut microbiome.

Previous evidence shows that the establishment of the gut microbiome in offspring is mainly based on vertical transmission of microbes (Nyangahu et al., 2018), and consequently, disruption of maternal gut microbiota is expected to exert negative impacts on immunity and sociability of offspring (Desbonnet et al., 2014). Moreover, antibiotic treatment on pregnant dams will lead to the aggravated immune response in offspring of antibiotic-treated females and may put them at higher risk for immune-mediated diseases (Miller et al., 2018). Therefore, preference for healthy females over those with dysbiosis to mate may benefit their offspring because disruption of maternal gut microbiota will change the immunity and sociability of offspring through vertical transmission of maternal microbes. Given that the gut microbiome may have a role in mating preference and mate choice, a balance of gut microbiota will be crucial for the sexual selection and reproduction of mammal species at the population level.

## Conclusion

To our best knowledge, we may present the first evidence that gut microbiota dysbiosis not only altered disease-related metabolic pathways but also reduced the sexual attractiveness of mice. The alteration of gut microbiota composition together with decreased health conditions may account for the reduction of sexual attractiveness of females. We argue that knowledge of the gut microbiome is fundamental to our understanding of the sexual attractiveness and reproductive strategies of mammals. Further understanding of the mechanisms underlying the relationships between gut microbiota, sexual selection, and mate choice will provide us with a new insight into the role of gut microbiota in shaping mating preference and reproduction strategies of mammals.

## Data Availability Statement

Data was deposited at https://doi.org/10.6084/m9.figshare.17708114.v1. Cecal metagenome sequence data generated and analyzed are available in the NCBI Sequence Read Archive under accession PRJNA831647.

## Ethics Statement

The animal study was reviewed and approved by Animal Experimentation Ethics Committee of Qufu Normal University.

## Author Contributions

XY designed the study and wrote the manuscript. MC collected the data. MC and XY did the analyses. Both authors contributed intellectually to the manuscript.

## Conflict of Interest

The authors declare that the research was conducted in the absence of any commercial or financial relationships that could be construed as a potential conflict of interest.

## Publisher’s Note

All claims expressed in this article are solely those of the authors and do not necessarily represent those of their affiliated organizations, or those of the publisher, the editors and the reviewers. Any product that may be evaluated in this article, or claim that may be made by its manufacturer, is not guaranteed or endorsed by the publisher.
