# Supplementary material for: Gut Dysbiosis Has the Potential to Reduce the Sexual Attractiveness of Mouse Female
Source: Front Microbiol. 2022 May 23;13:916766. doi: 10.3389/fmicb.2022.916766 (PMC9169628; doi:10.3389/fmicb.2022.916766)
Supplement: Supplementary file 1 [file Data_Sheet_1.docx]

**Table S1** Paired t test for female attractiveness to males in this study. “#” indicates the ID of each mouse. MC, MT, FC and FT refer to control males, antibiotic-treated males, control females, antibiotic-treated females, respectively. For all statistical tests on the contact times, travel distances and time spent, “df” is 18.

| Chooser | Paired female | | Paired t test | Chooser | Paired female | | Paired t test |
| --- | --- | --- | --- | --- | --- | --- | --- |
| MC♂ | FC♀ | FT♀ |  | MT♂ | FC♀ | FT♀ |  |
| 1^#^ | 1^#^ | 1^#^ | t = 1.830, P = 0.048  t = 3.129, P = 0.003  t = 3.136, P = 0.004 | 1^#^ | 6^#^ | 6^#^ | t = 1.830, P = 0.019  t = 3.129, P = 0.021  t = 3.136, P = 0.013 |
| 2^#^ | 2^#^ | 2^#^ |  | 2^#^ | 7^#^ | 7^#^ |  |
| 3^#^ | 3^#^ | 3^#^ |  | 3^#^ | 8^#^ | 8^#^ |  |
| 4^#^ | 4^#^ | 4^#^ |  | 4^#^ | 9^#^ | 9^#^ |  |
| 5^#^ | 5^#^ | 5^#^ |  | 5^#^ | 10^#^ | 10^#^ |  |
| 6^#^ | 1^#^ | 1^#^ |  | 6^#^ | 6^#^ | 6^#^ |  |
| 7^#^ | 2^#^ | 2^#^ |  | 7^#^ | 7^#^ | 7^#^ |  |
| 8^#^ | 3^#^ | 3^#^ |  | 8^#^ | 8^#^ | 8^#^ |  |
| 9^#^ | 4^#^ | 4^#^ |  | 9^#^ | 9^#^ | 9^#^ |  |
| 10^#^ | 5^#^ | 5^#^ |  | 10^#^ | 10^#^ | 10^#^ |  |

**Fig. S1.** A photo (a) and graphical presentation (b) of experimental design in this study. One control female (FC) and one antibiotic-treated female (FT) were placed in either of the two cages in each test session. The control males (MC) and antibiotic-treated males (MT) were tested individually.


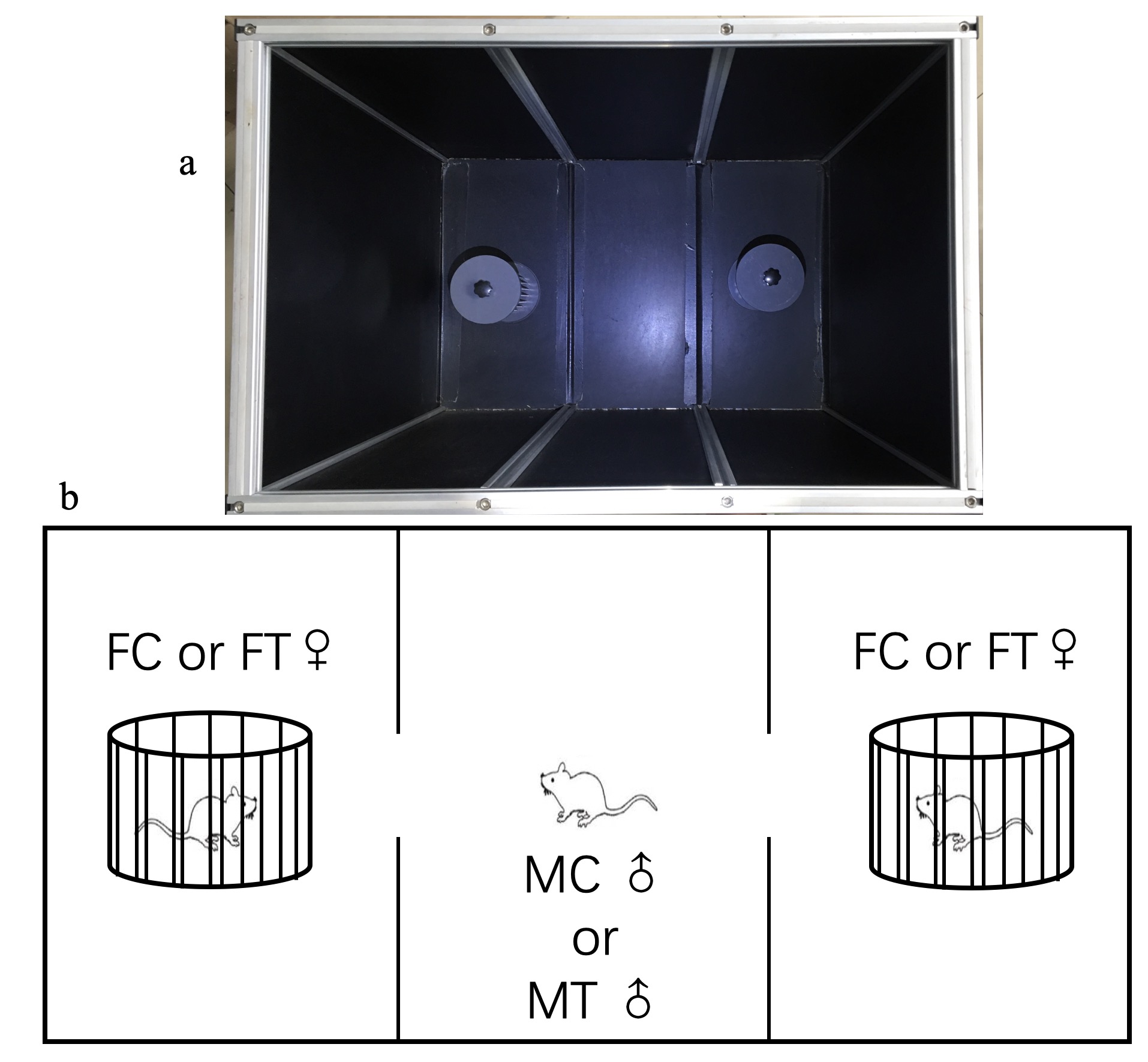


**Fig. S2.** Comparison of the α- diversity indices (a, b, c and d) and β-diversity of principal-coordinates analysis (PCoA) based on Bray-Curtis dissimilarities (e) of the gut microbiota of the control males (MC) and antibiotic-treated males (MT). Statistical significance: **, P < 0.01; ***, P < 0.001.


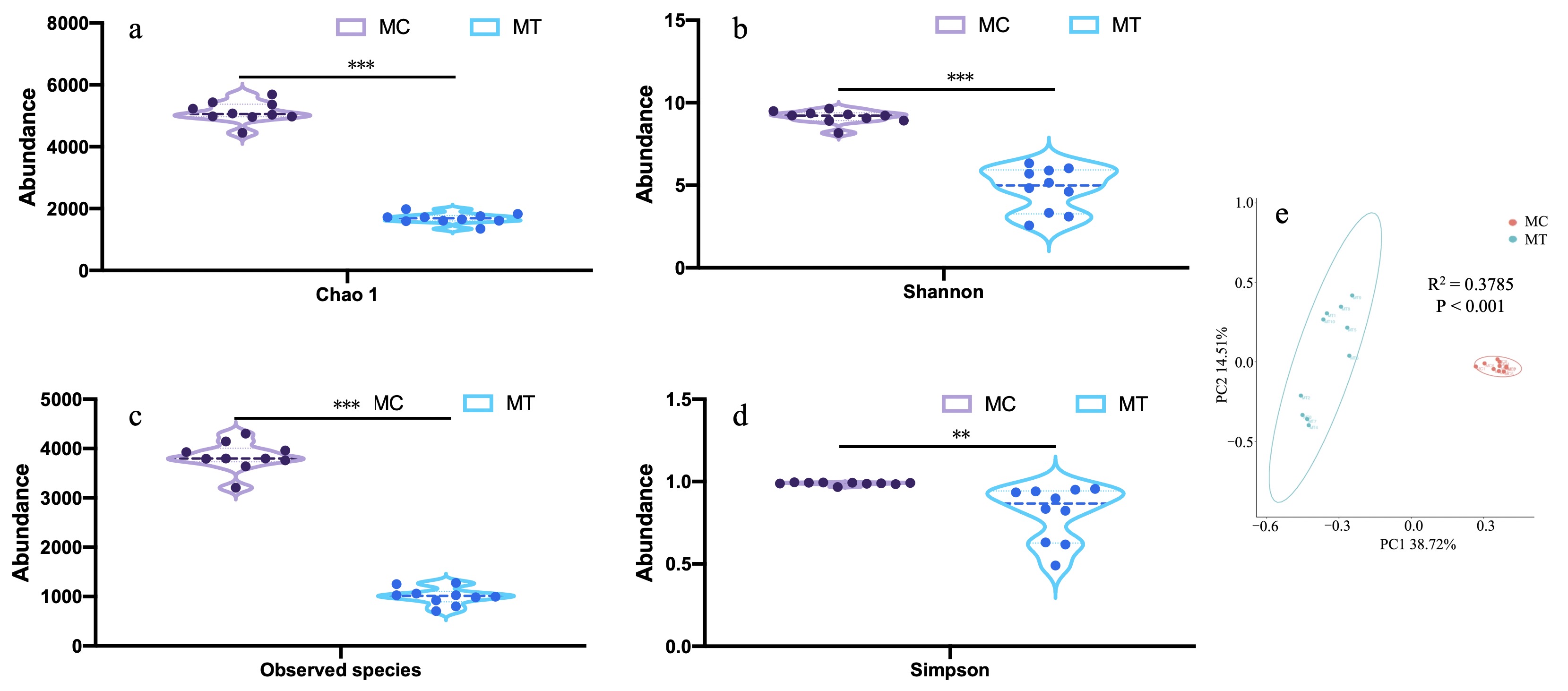


**Fig. S3.** Relative abundance of top 15 phyla (a) and 15 genera (b) that were significantly different between the control females (FC) and antibiotic-treated females (FT).

**Fig. S4.** Relative abundance of top 15 phyla (a) and 15 genera (b) that were significantly different between the control males (MC) and antibiotic-treated males (MT).


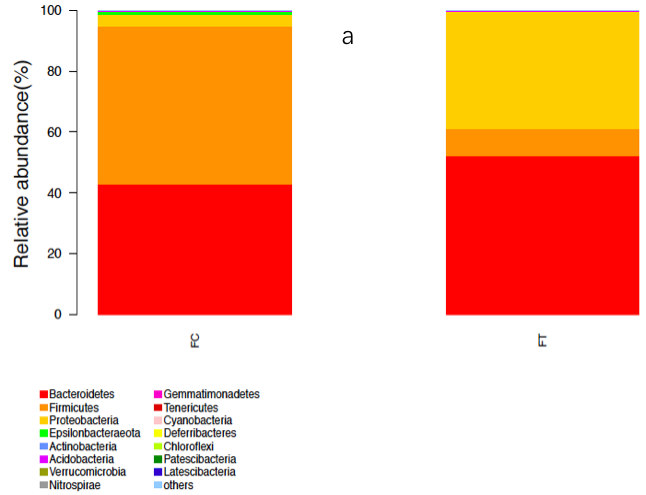


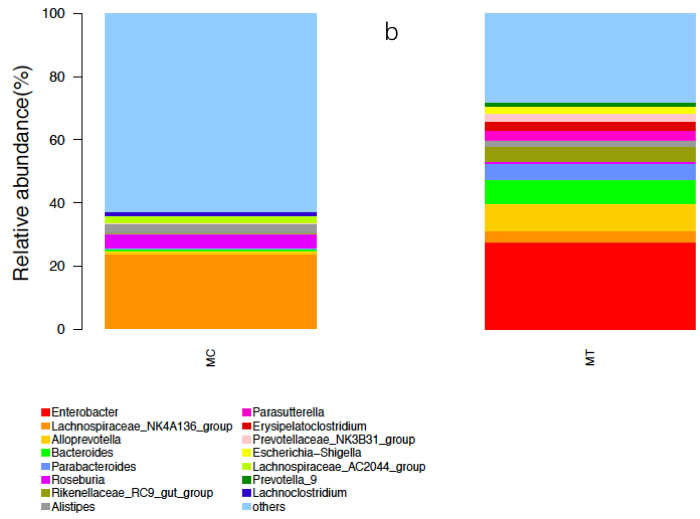


**Fig. S5.** Effects of antibiotic treatments on body weight of males (a) and females (b).
